# Molecular Characteristics of ST1193 Clone among Phylogenetic Group B2 Non-ST131 Fluoroquinolone-Resistant *Escherichia coli*

**DOI:** 10.3389/fmicb.2017.02294

**Published:** 2017-11-21

**Authors:** Jing Wu, Fangjun Lan, Yanfang Lu, Qingwen He, Bin Li

**Affiliations:** ^1^Department of Rheumatology and Clinical Immunology, Zhujiang Hospital of Southern Medical University, Guangzhou, China; ^2^Department of Clinical Laboratory, Fujian Medical University Union Hospital, Fuzhou, China; ^3^Department of Clinical Laboratory, Maternal and Children’s Health Hospital of Fujian Province, Fuzhou, China

**Keywords:** *Escherichia coli*, ST1193, fluoroquinolone resistance, phylogenetic group B2, O75

## Abstract

**Objectives:** Sequence type 1193 is emerging as a new, virulent and resistant lineage among fluoroquinolone resistant *Escherichia coli* (FQ^r^ *E. coli*). In this study, we investigated the prevalence and molecular characteristics of this clone isolated from a Chinese university hospital.

**Methods:** 73 phylogenetic group B2-FQ^r^-non-ST131 isolates were collected from August 2014 and August 2015 at a Chinese university hospital. Isolates were screened for ST1193 by multilocus sequence typing. *E. coli* ST1193 then underwent lactose fermentation determination, susceptibility testing, virulence genotyping, PCR-based O typing, pulsed-field gel electrophoresis (PFGE) and FQ^r^ mechanism analysis.

**Results:** Of 73 B2-FQ^r^-non ST131 *E. coli* isolates, 69.9% (*n* = 51) were ST1193. 90.2% (46/51) of ST1193 isolates were O75 serotype and 96.1% (49/51) of the ST1193 isolates were lactose non-fermenters. 35 clusters were identified by PFGE. ST1193 isolates exhibited a set of 3 conserved mutations defining quinolone-resistance determining region substitutions (*gyrA* S83L, D87N, and *parC* S80I). The most frequent VF genes detected in these *E. coli* ST1193 isolates were *fyuA* (yersiniabactin, 96.1%), *fimH* (type 1 fimbriae, 94.1%), *iutA* (iron uptake gene, 90.2%), *kpsMT II* (group II capsule, 90.2%), *kpsK1* (K1 capsule, 86.3%) and PAI.

**Conclusion:** ST1193 lineage accounts for the majority of group B2-FQ^r^-non-ST131 *E. coli* clinical isolates. Most of the ST1193 are serotype O75 and lactose non-fermenting. Strategic surveillance and control schemes are needed in the future for this newly emerging clone of *E. coli*: B2-FQ^r^-ST1193.

## Introduction

*Escherichia coli* (*E. coli*) is commonly found in the gut flora of humans and can cause many different kinds of extra-intestinal infections, such as septicemias, meningitis and urinary tract infections (Pitout, 2012; Poolman and Wacker, 2016). Many methods can be used for typing *E. coli*, including serotyping, pulsed-field gel electrophoresis (PFGE) and various PCR-based molecular methods(Jonas et al., 2003; Cespedes et al., 2017; Roer et al., 2017). Multilocus sequence typing (MLST) is a PCR-based molecular typing method and is considered the “gold standard” subtyping technique that can usually be used to detect the genetic relatedness between strains and identify a specific clone with high discriminatory ability for various bacterial pathogens (Maiden et al., 2013; Perez-Losada et al., 2013). The method is now widely used in the identification and classification of *E. coli*. Successful MLST clones of *E. coli* have been reported in various regions of the world (Nicolas-Chanoine et al., 2014; Dautzenberg et al., 2016). *E. coli* ST131 is a classic clonal group from phylogenetic group B2. It typically possesses a wide variety of virulence factors (VFs) and is strongly associated with resistance to fluoroquinolone and extended spectrum cephalosporins (Nicolas-Chanoine et al., 2014; Dautzenberg et al., 2016).

Simultaneously, ST1193 has been reported recently, as a new, virulent and resistant *E. coli* that commonly causes extra-intestinal infections (Platell et al., 2012; Chang et al., 2014; Matsumura et al., 2017). *E. coli* ST1193 shares many common features, including fluoroquinolone resistance (FQ^r^), lactose non-fermenting and phylogenetic group B2 (Platell et al., 2012; Chang et al., 2014). In China, *E. coli* ST1193 has been sporadically reported (Zhao et al., 2015; Xia et al., 2017). However, no data was available as to the detailed characteristics of the lineage in this region. The aim of this study was to detect the prevalence and molecular characteristics of ST1193 among phylogenetic group B2-FQ^r^-non-ST131 *E. coli* in China.

## Materials and Methods

### Bacterial Isolates

A total of 700 non-duplicate *E. coli* clinical isolates were consecutively collected at a Chinese university hospital (Fujian Medical University Union Hospital, Fuzhou, China) between August 2014 and August 2015. 250 phylogenetic group B2 isolates were identified using PCR method as previously described (Clermont et al., 2013). Group B2 isolates were then tested for susceptibility to ciprofloxacin (CIP) by disk diffusion test according to CLSI (CLSI, 2017). B2-FQ^r^ *E. coli* isolates then underwent the PCR-based method for ST131-associated SNPs in *mdh* and *gyrB* as previously described (Johnson et al., 2009).

### MLST and Further Isolate Selection

B2-FQ^r^-non-ST131 *E. coli* isolates underwent MLST according to the Achtman scheme using seven housekeeping genes (*adk*, *fumC*, *gyrB*, *icd*, *mdh*, *purA*, and *recA*)^1^. *E. coli* ST1193 isolates were further characterized in detail below in this study.

### Susceptibility Testing and Lactose Fermentation Determination

Antimicrobial susceptibility of *E. coli* ST1193 isolates was determined by disk diffusion test to nine antimicrobials, which are commonly used in clinical therapy. The antibiotics tested in this study were as follows: aztreonam (30 μg), cefotaxime (30 μg), ceftazidime (30 μg), cefepime (30 μg), ertapenem (10 μg), imipenem (10 μg), piperacillin-tazobactam (100/10 μg), amikacin (30 μg), and trimethoprim-sulfamethoxazole (1.25/23.75 μg). All disks were obtained from Oxide, Ltd (Cambridge, United Kingdom). The results were interpreted according to the breakpoints of the CLSI criteria (CLSI, 2017). *E. coli* ATCC 25922 was used for routine quality control. Lactose fermentation was determined using KIA (Kligler’s Iron Agar).

### O Typing

Molecular serotyping of *E. coli* ST1193 isolates was performed by a multiplex PCR method to detect 16 serogroups, including O1, O2, O4, O6, O7, O8, O12, O15, O16, O18, O21, O22, O25, O75, O83, and O157 (DebRoy et al., 2011). Isolates that could not be typed using this method were classified as non-typeable in this study.

### Pulsed-Field Gel Electrophoresis

In order to determine the genetic relateness of the *E. coli* ST1193 isolates, isolates were subjected to PFGE analysis using XbaI digestion as previously described (Li et al., 2011). A PFGE dendrogram was constructed with BioNumerics software (Applied Maths, Sint-Martens-Latem, Belgium) according to the unweighted pair group method based on Dice coefficients. Isolates with a Dice similarity index ≥85% were considered to belong to the same PFGE group (Gibreel et al., 2012; Hussain et al., 2012).

### Virulence Gene Profiling

The presence of 26 known VFs genes was screened by a multiplex PCR method (Johnson and Stell, 2000). For each isolate, the virulence score (VF score) was calculated as the sum of all virulence-associated genes detected in this study. The sum of all the VF scores of the isolates was then calculated, and finally this sum was divided by the number of isolates to give the mean VF score.

### Detection of Fluoroquinolone Resistance Genes and *bla*_CTX-M_ Gene

*Escherichia coli* ST1193 isolates resistant to cefotaxime and/or ceftazidime underwent detection of *bla*_CTX-M_ using PCR and sequencing (Li et al., 2011). As to all ST1193 isolates, mutations in the quinolone-resistance determining region (QRDR) in chromosomal *gyrA*, *parC* and *parE* genes were determined by PCR and sequencing (Everett et al., 1996). The presence of plasmid mediated quinolone resistance determinants [PMQRs; *qnrA*, *qnrB*, *qnrC*, *qnrD*, *qnrS*, and *aac(6′)-Ib-cr*] were detected by PCR as previously described (Chen et al., 2012).

## Results

### Bacterial Isolates

One hundred and thirty one B2-FQ^r^ *E. coli* isolates were collected in this study, including 58 ST131 and 73 non-ST131 strains. 73 B2-FQ^r^-non ST131 *E. coli* isolates were grouped into 10 distinct STs, with the predominant ST being ST1193 (*n* = 51, 69.9%), followed by ST95 (*n* = 4, 5.5%), ST140 (*n* = 3, 4.1%), ST648 (*n* = 3, 4.1%), ST73 (*n* = 3, 4.1%), ST92 (*n* = 2, 2.7%), ST117 (*n* = 2, 2.7%), ST354 (*n* = 2, 2.7%), ST1318 (*n* = 1, 1.4%), ST2558 (*n* = 1, 1.4%), and ST3177 (*n* = 1, 1.4%). The majority of *E. coli* ST1193 strains (34, 66.7%) were isolated from urine, followed by blood (*n* = 7, 13.7%), secretions (*n* = 6, 11.8%), fluids (*n* = 3, 5.9%), and pus (*n* = 1, 2.0%). Females accounted for 72.5% (37/51) of the ST1193 isolates, and the average age was 65 years (range: 10–91 years). *E. coli* ST1193 strains were isolated from different wards (Supplementary Table S1).

### O Typing and Lactose Fermentation Determination

90.2% (46/51) of the *E. coli* ST1193 were classified as O-type O75. The other five isolates could not be typed. 96.1% (49/51) of the ST1193 isolates were lactose non-fermenters, including 46 O75 and 3 non-O typeable isolates.

### Antimicrobial Susceptibility Testing

*Escherichia coli* ST1193 isolates showed high resistance against trimethoprim-sulfamethoxazole (64.7%). Resistance rates were low than 50% to cefotaxime (45.1%), aztreonam (21.6%), cefepime (19.6%), ceftazidime (9.8%), and amikacin (7.8%), respectively. All the strains were susceptible to imipenem and ertapenem.

### Bacterial Clonal Relatedness

**FIGURE 1:**
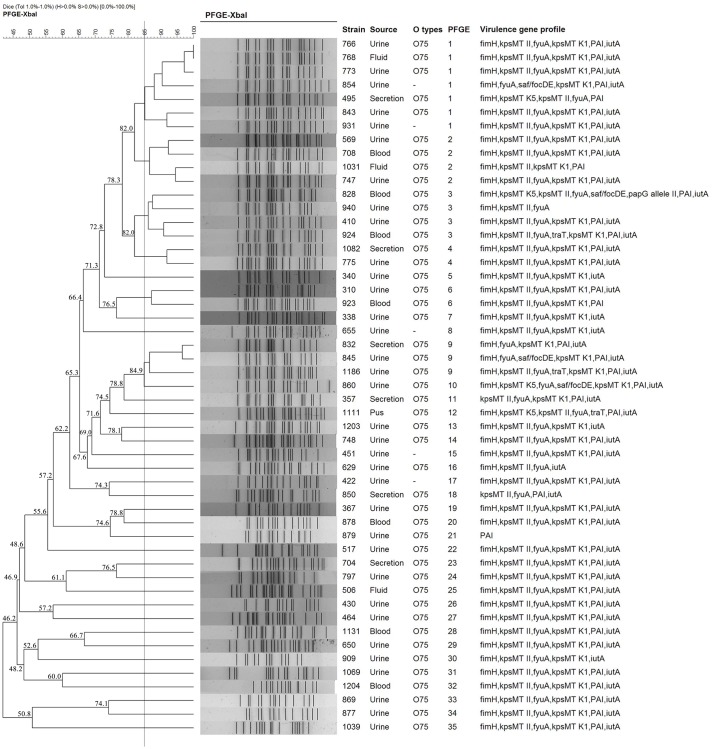
Dendrogram of the PFGE patterns of chromosomal DNA restriction fragments from 51 ST1193 *Escherichia coli* isolates. The dendrogram was constructed using the UPGMA method. Dice coefficients (percentages) are listed in the scale on the top of the dendrogram. Strain number, sample source, O serotype, PFGE patterns and virulence factors (VFs) are included.

Pulsed-field gel electrophoresis patterns of the 51 *E. coli* ST1193 isolates identified 35 PFGE types (clusters 1–35) (**Figure 1**). No single dominant intra-hospital PFGE type was detected when using a cutoff of 85% similarity. Three PFGE subtypes (clusters 1, 2, and 3) encompassed at least three ST1193 isolates.

### Fluoroquinolone Resistance Characteristics and *bla*_CTX-M_ Gene

All of the 51 *E. coli* ST1193 isolates contained a set of 3 conserved mutations defining QRDR substitutions (*gyrA* S83L, D87N, and *parC* S80I). Two ST1193 isolates had additional mutations in the QRDR of *parC* (N167Y). No mutation was found in *parE*. Meanwhile, two isolates harbored *aac(6′)-Ib-cr* variant and none of other PMQR determinants (*qnrA*, q*nrB*, *qnrC*, *qnrD*, *qnrS*, oqxAB, and *qepA*) were detected in this study.

Among 23 cefotaxime and/or ceftazidime *E. coli* ST1193 isolates, 22 (95.7%) contained *bla*_CTX-M_ genes. Of these *bla*_CTX-M_ positive isolates, 12 isolates carried *bla*_CTX-M-14_, 6 isolates carried *bla*_CTX-M-15_ and 2 isolates carried *bla*_CTX-M-123_. The remaining 2 isolates co-harbored *bla*_CTX-M-14_ and *bla*_CTX-M-15_. *bla*_CTX-M-2,_ *bla*_CTX-M-8_ and *bla*_CTX-M-25_ groups were not detected in these isolates.

### Virulence Gene Profiling

The most frequent VF genes were *fyuA* (yersiniabactin, 96.1%), *fimH* (type 1 fimbriae, 94.1%), *iutA* (iron uptake gene, 90.2%), *kpsMT II* (group II capsule, 90.2%), *kpsK1* (K1 capsule, 86.3%) and PAI (86.3%), each of which was detected in ≥80% of the isolates. Four genes were identified in less than 10% of isolates, including *kpsK5* (7.8%), *sfa/focDE* (7.8%), *traT* (5.9%), and *papG* allele II (2.0%). *kpsMTIII*, *papAH*, *papEF*, *papC*, *papG alleleI*, *nfaE*, *rfc*, *papGII/III*, *hlyA*, *sfaS*, *gafD*, *cvaC*, *cdtB*, *focG*, *cnf1*, and *afa/draBC* were not detected in this study.

## Discussion

Fluoroquinolones are the most widely used antimicrobial agents for the treatment of different infections in China. The high frequency of fluoroquinolone resistance in *E. coli* is considered as a feature of clinical bacteriology in the last 10 years in China according to a CHINET report (Hu et al., 2016). Fluoroquinolone resistance usually occurred in low-virulence *E. coli* phylogenetic group A isolates rather than in isolates from groups B2 and D. However, the recent worldwide emergence of B2-FQ^r^ -*E. coli* has occurred primarily through clonal expansion of *E. coli* ST131 (Nicolas-Chanoine et al., 2014; Dautzenberg et al., 2016). Our study confirmed that while ST131 is still the predominant subclone among B2-FQ^r^ -*E. coli* clinical isolates in China, ST1193 is emerging as an important subgroup and accounts for a similar percentage with ST131 among group B2-FQ^r^-*E. coli*. The reasons for the expansion of such subclone are unclear and it is worth investigating in future study to explain its dissemination in China.

Historically, *E. coli* serotype O75 isolates are strongly associated with urinary tract infection and sepsis in humans, and virulence-associated phylogenetic group B2 (Devine et al., 1989; Obata-Yasuoka et al., 2002; Platell et al., 2012). Moreover, O75 strains are usually non-lactose fermenter (Platell et al., 2012; Chang et al., 2014). Our results were consistent with the results from previous studies as the majority of our ST1193 isolates were non-lactose fermenter (96.1%) and belonged to serotype O75 (90.2%). Because O75 isolates were never found to be associated with FQ resistance in previous studies, the recent emergence of O75-B2-FQ^r^-ST1193 *E. coli* isolates are probably due to clonal expansion (Platell et al., 2012).

In this study, ciprofloxacin resistance was mainly due to the accumulation of three point mutations in DNA gyrase or topoisomerase IV (*gyrA* S83L, D87N, and *parC* S80I), which are the primary bacterial target of quinolone (Hooper and Jacoby, 2015; Correia et al., 2017). The pattern of non-synonymous mutations found in our B2-FQ^r^-ST1193 isolates was identical to those observed in Australian and Korean ST1193 isolates (Platell et al., 2012; Chang et al., 2014). As discussed in previous study (Platell et al., 2012), the homogeneity of ST1193 isolates suggests this subclone emerged and spreaded from a common ancestor. Meanwhile, previous study suggested that more distinctive QRDR mutations probably provided *E. coli* ST131 *H*30 isolates with fitness advantages over other fluoroquinolone resistant *E. coli* isolates (Johnson et al., 2015). In this study, all the *E. coli* ST1193 isolates contained the same distinctive combination of three non-synonymous mutations (*gyrA* S83L, D87N, and *parC* S80I). These three QRDR mutations found in ST1193 isolates are the only ones that have been experimentally proved to increase fluoroquinolone MICs at the present time (Marcusson et al., 2009). Therefore, non-synonymous QRDR mutations in *gyrA* and *parC* may play a similar role in the fitness of *E. coli* ST1193 isolates as what they do in ST131 lineage.

The clonal dissemination of *bla*CTX-M-55 among *E. coli* isolates causing urinary tract infections maybe due to the expansion of ST1193 clone in one previous study (Xia et al., 2017). In this study, we attempted to identify the *bla*CTX-M genes in our third generation cephalosporin resistant ST1193 isolates. Although our results showed a high percentage (95.7%) of third generation cephalosporin resistant isolates harboring different *bla*CTX-M genes, no correlation between ST1193 clone and any special *bla*CTX-M genes was found in this study. Regional difference may be the main reason for the disparity of these two studies. Further nationwide study should be carried out to explain the difference in China.

Identical virulence gene profiles as one common phenotypic characteristic were presenting in previous studies (Platell et al., 2012; Cremet et al., 2013). In this study, we also exhibited identical virulence genes profile (*fimH*, *fyuA*, *iutA*, *kpsMT II*, *kpsK1*, and PAI) among most of the ST1193 isolates. Together with the results of serotyping, fluoroquinolone resistance and phylogenetic group B2, the high level of homogeneity between ST1193 strains from different geographical regions suggests a probable divergence from a common ancestor.

Meanwhile, we identified a total of 35 PFGE types using PFGE analysis in this study, and no dominant intra-hospital PFGE type was found using a cutoff of 85% similarity. Our results suggested that *E. coli* ST1193 clinical isolates were not spread by clonal strains in our hospital.

## Conclusion

ST1193 lineage account for the majority of B2-FQ^r^-non-ST131 *E. coli* population in this study. These ST1193 isolates are highly clonal diversity as indicated by PFGE patterns. Most of the ST1193 isolates were serotype O75 and lactose non-fermenting. Strategic surveillance and control schemes are needed in the future for this newly emerging clone of *E. coli*: B2-FQ^r^-ST1193.

## Ethics Statement

This study was approved by The Institutional Review Board of the Fujian Medical University Union Hospital, Fuzhou, China. No consent was needed for this study.

## Author Contributions

JW, FL, YL, and QH performed experiments. JW and FL conceived the study and analyzed the results. BL supervised the study and prepared the manuscript. All authors read and approved the final manuscript.

## Conflict of Interest Statement

The authors declare that the research was conducted in the absence of any commercial or financial relationships that could be construed as a potential conflict of interest.

## Footnotes

1 http://mlst.ucc.ie/mlst/dbs/Ecoli
