# Supplementary material for: Molecular Characteristics of ST1193 Clone among Phylogenetic Group B2 Non-ST131 Fluoroquinolone-Resistant Escherichia coli
Source: Front Microbiol. 2017 Nov 21;8:2294. doi: 10.3389/fmicb.2017.02294 (PMC5702334; doi:10.3389/fmicb.2017.02294)
Supplement: Supplementary file 1 [file Table_1.DOCX]

Table S1. The main characteristics of 51 *E. coli* ST1193 isolates.

| No. | strains | Isolated date | sex | age | wards | lactose  fermentation | O  serotype | Quinolone resistance (chromosome) | | Resistance profiles |
| --- | --- | --- | --- | --- | --- | --- | --- | --- | --- | --- |
|  |  |  |  |  |  |  |  | gyrA | parC |  |
| 1 | EC310 | 2014.8 | F | 72 | endocrinology | + | O75 | S83L,D87N | S80I | FEP,CTX,ATM,AK |
| 2 | EC338 | 2014.8 | F | 59 | emergency | + | O75 | S83L,D87N | S80I | AK |
| 3 | EC340 | 2014.8 | F | 63 | neurology | + | O75 | S83L,D87N | S80I | SXT |
| 4 | EC357 | 2014.8 | F | 46 | gynaecology | + | O75 | S83L,D87N | S80I | - |
| 5 | EC367 | 2014.8 | F | 49 | urology surgery | + | O75 | S83L,D87N | S80I | FEP,CTX,CAZ,ATM,SXT |
| 6 | EC410 | 2014.9 | M | 66 | hematology | + | O75 | S83L,D87N | S80I | SXT |
| 7 | EC422 | 2014.9 | F | 61 | emergency | + | - | S83L,D87N | S80I | SXT |
| 8 | EC430 | 2014.9 | F | 62 | endocrinology | + | O75 | S83L,D87N | S80I | FEP,CTX,ATM,AK |
| 9 | EC451 | 2014.9 | F | 82 | oncology | - | - | S83L,D87N | S80I | CTX,SXT |
| 10 | EC464 | 2014.10 | F | 46 | hematology | + | O75 | S83L,D87N | S80I,N167Y | SXT |
| 11 | EC495 | 2014.10 | M | 56 | colorectal surgery | + | O75 | S83L,D87N | S80I | SXT |
| 12 | EC506 | 2014.11 | M | 62 | colorectal surgery | + | O75 | S83L,D87N | S80I | CTX |
| 13 | EC517 | 2014.11 | F | 52 | hepatic-biliary surgery | + | O75 | S83L,D87N | S80I | - |
| 14 | EC569 | 2014.11 | F | 51 | hematology | + | O75 | S83L,D87N | S80I | CTX,SXT |
| 15 | EC629 | 2014.11 | M | 47 | neurosurgery | + | O75 | S83L,D87N | S80I,N167Y | CTX |
| 16 | EC650 | 2014.12 | F | 59 | nephrology | + | O75 | S83L,D87N | S80I | - |
| 17 | EC655 | 2014.12 | F | 68 | colorectal surgery | + | - | S83L,D87N | S80I | SXT |
| 18 | EC704 | 2015.1 | F | 59 | emergency | + | O75 | S83L,D87N | S80I | CTX,SXT |
| 19 | EC708 | 2015.1 | F | 52 | emergency | + | O75 | S83L,D87N | S80I | CTX |
| 20 | EC747 | 2015.1 | F | 78 | orthopedics | + | O75 | S83L,D87N | S80I | SXT |
| 21 | EC748 | 2015.1 | F | 56 | neurology | + | O75 | S83L,D87N | S80I | - |
| 22 | EC766 | 2015.1 | F | 79 | cadiology | + | O75 | S83L,D87N | S80I | - |
| 23 | EC768 | 2015.1 | M | 47 | colorectal surgery | + | O75 | S83L,D87N | S80I | - |
| 24 | EC773 | 2015.1 | F | 75 | orthopedics | + | O75 | S83L,D87N | S80I | - |
| 25 | EC775 | 2015.1 | F | 72 | colorectal surgery | + | O75 | S83L,D87N | S80I | CTX,AK,SXT |
| 26 | EC797 | 2015.1 | F | 63 | nephrology | + | O75 | S83L,D87N | S80I | CTX |
| 27 | EC828 | 2015.1 | F | 31 | hematology | + | O75 | S83L,D87N | S80I | FEP,CTX,CAZ,ATM,SXT |
| 28 | EC832 | 2015.1 | M | 32 | hematology | + | O75 | S83L,D87N | S80I | FEP,CTX,ATM,SXT |
| 29 | EC843 | 2015.1 | M | 91 | senior officials inpatient ward | + | O75 | S83L,D87N | S80I | CTX |
| 30 | EC845 | 2015.1 | F | 65 | endocrinology | + | O75 | S83L,D87N | S80I | SXT |
| 31 | EC850 | 2015.1 | M | 34 | thyroid | + | O75 | S83L,D87N | S80I | SXT |
| 32 | EC854 | 2015.1 | F | 60 | nephrology | - | - | S83L,D87N | S80I | FEP,CTX,CAZ,ATM,SXT |
| 33 | EC860 | 2015.1 | F | 41 | emergency | + | O75 | S83L,D87N | S80I | SXT |
| 34 | EC869 | 2015.2 | F | 50 | endocrinology | + | O75 | S83L,D87N | S80I | SXT |
| 35 | EC877 | 2015.2 | F | 86 | senior officials inpatient ward | + | O75 | S83L,D87N | S80I | FEP,CTX,CAZ,ATM,SXT |
| 36 | EC878 | 2015.3 | F | 86 | hematology | + | O75 | S83L,D87N | S80I | SXT |
| 37 | EC879 | 2015.3 | F | 53 | senior officials inpatient ward | + | O75 | S83L,D87N | S80I | FEP,CTX,CAZ,ATM,SXT |
| 38 | EC909 | 2015.3 | M | 50 | colorectal surgery | + | O75 | S83L,D87N | S80I | - |
| 39 | EC923 | 2015.4 | M | 38 | emergency | + | O75 | S83L,D87N | S80I | CTX,SXT |
| 40 | EC924 | 2015.4 | M | 19 | hematology | + | O75 | S83L,D87N | S80I | FEP,CTX,ATM,SXT |
| 41 | EC931 | 2015.4 | F | 52 | nephrology | - | - | S83L,D87N | S80I | - |
| 42 | EC940 | 2015.4 | F | 61 | urology surgery | + | O75 | S83L,D87N | S80I | CTX,ATM,SXT |
| 43 | EC1031 | 2015.5 | M | 20 | emergency | + | O75 | S83L,D87N | S80I | SXT |
| 44 | EC1039 | 2015.5 | F | 68 | cadiology | + | O75 | S83L,D87N | S80I | FEP,CTX,ATM,SXT |
| 45 | EC1069 | 2015.5 | F | 65 | colorectal surgery | + | O75 | S83L,D87N | S80I | SXT |
| 46 | EC1082 | 2015.6 | F | 42 | gynaecology | + | O75 | S83L,D87N | S80I | CTX,SXT |
| 47 | EC1111 | 2015.6 | F | 10 | paediatrics | + | O75 | S83L,D87N | S80I | SXT |
| 48 | EC1186 | 2015.6 | F | 76 | gastroenterology surgery | + | O75 | S83L,D87N | S80I | - |
| 49 | EC1131 | 2015.6 | M | 56 | emergency | + | O75 | S83L,D87N | S80I | CTX,SXT |
| 50 | EC1203 | 2015.6 | F | 58 | endocrinology | + | O75 | S83L,D87N | S80I | SXT |
| 51 | EC1204 | 2015.8 | M | 62 | emergency | + | O75 | S83L,D87N | S80I | SXT |
